# Aptamers Targeting IL17A and Its Receptor Suppress IL17 Signaling in Different Cell Types

**DOI:** 10.3390/ph19020238

**Published:** 2026-01-29

**Authors:** Anastasiya Solovieva, Dariya Rippinen, Anna Davydova, Veronika Goncharova, Vladimir Koval, Mariya Vorobyeva, Maksim Korolev

**Affiliations:** 1 Research Institute of Clinical and Experimental Lymphology, Affiliated Branch of Federal Research Center of Cytology and Genetics, Siberian Division of the Russian Academy of Sciences, 630117 Novosibirsk, Russia; d.rippinen@icloud.com (D.R.); v.goncharova@icgbio.ru (V.G.); koval.v.v97@gmail.com (V.K.); kormax@bk.ru (M.K.); 2 Institute of Chemical Biology and Fundamental Medicine, Siberian Division of Russian Academy of Sciences, 630090 Novosibirsk, Russia; anna.davydova@niboch.nsc.ru

**Keywords:** interleukin-17A (IL-17A), IL-17 receptor, aptamer, spondyloarthritis, rheumatoid arthritis, fibroblast-like synoviocytes, inflammation

## Abstract

**Background/Objectives**: Interleukin-17A (IL-17A) is a key pathogenic cytokine in autoimmune arthropathies. Current monoclonal antibody inhibitors targeting the IL-17/IL-17RA axis demonstrate clinical efficacy but face significant limitations, including immunogenicity, the loss of therapeutic response, and cold-chain storage. Our study evaluated oligonucleotide aptamers targeting IL-17A and its receptor as an alternative to monoclonal antibodies to suppress an IL-17A-induced inflammatory response in cell models relevant to immunoinflammatory rheumatic diseases. **Methods**: We examined three aptamers: 2′-F-RNA aptamers Apt21-2 and Apt3-4 specific to IL-17A and DNA aptamer RA10-6 targeting the receptor of IL-17A. Their ability to suppress IL-17A functional activity was assessed in peripheral blood mononuclear cells (PBMCs) from healthy donors and personalized fibroblast-like synoviocytes (FLSs) from patients with axial spondyloarthritis (axSpA) and rheumatoid arthritis (RA). Inhibition was measured by quantifying IL-6 and MMP-13 secretion using ELISA and flow cytometry, using secukinumab as a reference control. **Results**: In PBMC, all aptamers suppressed IL-17A-stimulated IL-6 secretion and cell proliferation in a concentration-dependent manner (17–200 nM), with a 65–85% efficacy, comparable to that of secukinumab. In axSpA-derived FLS, we observed time-dependent efficacy: At 4 h, all three aptamers suppressed IL-6 to the same extent as secukinumab; at 24 h, RA10-6 maintained high efficacy while Apt21-2 and Apt3-4 showed reduced activity. A combination of receptor-targeting RA10-6 with anti-IL-17A aptamers resulted in synergistic IL-6 suppression. All aptamers reduced MMP-13 to basal levels. RA-derived FLS showed diminished responses to all inhibitors. **Conclusions**: Aptamers demonstrate high specificity and sustained efficacy in suppressing IL-17A signaling for an in vitro model of spondyloarthritis, with superior performance over antibodies. Disease-dependent differential efficacy in RA FLS reflects heterogeneity consistent with limited clinical anti-IL-17 efficacy in RA. These findings show the strong potential of the studied aptamers as an alternative to monoclonal antibodies for IL-17-associated inflammatory arthropathies, particularly spondyloarthritis.

## 1. Introduction

The IL-17 cytokine family, particularly IL-17A, plays a key role in the pathogenesis of a wide spectrum of diseases, including rheumatic, dermatological, and pulmonological ones, and ophthalmopathies [1,2,3]. Their imbalance leads to chronic inflammation with subsequent irreversible tissue damage [3]. In particular, in spondyloarthritides, an IL-17A hyperproduction causes progressive joint damage, enthesitis, and uveitis, resulting in disability of 13–45% of the patients in 8–20 years [2,4,5,6,7,8]. In psoriasis and psoriatic arthritis, IL-17A provokes chronic skin and joint inflammation, thus worsening life quality and working capacity [1,4,9]. In rheumatoid arthritis, IL-17 enhances the secretion of IL-6 and other inflammatory cytokines, causing proliferation and pannus formation. Moreover, the synergistic action of IL-17 and TNFα results in strengthening inflammation and tissue injury, erosive lesions, and cartilage destruction [10]. These pathologies are known for their chronic character and high public healthcare burden, while the timely use of effective anti-inflammatory treatment decreases inflammation activity, preserving the patient’s functional status and lowering social and economic costs [11,12].

One of the most effective therapeutic approaches for treating IL-17-associated diseases involves direct or indirect targeting of the cytokine or its receptors [2]. This strategy showed the greatest success in the treatment of spondyloarthritis, psoriatic arthritis, and psoriasis. To date, a range of monoclonal antibodies have been approved for their treatment, namely IL-17A inhibitors secukinumab, ixekizumab, and netakimab, IL-17RA inhibitor brodalumab, and the bispecific bimekizumab that blocks both IL-17A and IL-17F [2,13]. While IL-17 also impacts the pathogenesis of rheumatoid arthritis, anti-IL17 mAbs showed only moderate efficacy in RA patients, probably due to heterogeneity of RA, the presence of ‘synovial phenotypes’, and the synergistic action of inflammatory cytokines in RA [14,15].

Despite proven high clinical efficacy and a sufficient safety profile, the use of therapeutic mAbs faces certain limitations. Their immunogenicity and generation of neutralizing antibodies lead to the loss of therapeutic response and elevate the risk of adverse effects [16,17,18], while cold chain maintenance and parenteral injections limit treatment availability [19]. These factors stimulated the development of novel drug candidates, such as RORγt inhibitors, suppressing Th17 cell differentiation [20,21,22], or the anti-IL-17A affibody, Isokibep, which showed promising results for psoriasis and psoriatic arthritis during preclinical and clinical investigations ([23,24], NCT05623345).

Alternatively, therapeutic nucleic acids can be used for suppressing IL-17 signaling. Among them, oligonucleotide aptamers look very promising since they employ the same mode of action as monoclonal antibodies, while possessing a set of unique advantages [25,26]. Their main features include low immunogenicity, stable and reproducible chemical synthesis, and high tolerance to transportation and storage conditions, which is especially important in view of potential clinical application [26,27]. Multiple studies report that anti-inflammatory aptamers significantly reduce inflammation in cell and animal models of inflammatory joint and bone diseases [28,29,30,31]. In particular, aptamers against IL-4Rα, IL-6, and TNF-α showed comparable efficacy to established biologics in reducing tissue inflammation and disease symptoms [28].

In the present study, we evaluated the potential of oligonucleotide aptamers with respect to IL-17A and its receptor to suppress IL-17 signaling in inflammatory rheumatic diseases, namely axial spondyloarthritis and rheumatoid arthritis, using human-related cell models of these pathologies. Several anti-IL17A and anti-IL17RA have been reported [32,33,34,35]; among them, we chose anti-IL17A 2′-fluoro-RNA aptamers 3-4 and 21-2 with sub-nM binding affinity [33] and anti-IL17RA DNA aptamer RA10-6 with 1 nM K_D_ for receptor binding [32]. These aptamers demonstrated their ability to influence IL-17 signaling on murine models of chronic joint inflammation, osteoarthritis, and glucose-6-phosphate-induced arthritis [32,33]. Therefore, we found them most promising for evaluation on human arthritis models.

We examined the aptamers in vitro using two cell models: PBMC from a healthy donor and personalized cultures of fibroblast-like synoviocytes (FLSs) from patients with axSpA and seropositive RA. Here, we studied for the first time the ability of aptamers to influence IL-17 signaling in patient-derived cells relevant to axial spondyloarthritis and rheumatoid arthritis.

## 2. Results

### 2.1. Targeted Inhibition of IL-17A Functional Activity by Oligonucleotide Aptamers on PBMC Model

The cell culture was supplied by recombinant IL-17A at a concentration of 50 ng/mL that reliably elevates the PBMC amount by 55.9 ± 8.2% and the level of IL-6 secretion. As a control of specificity, we employed a non-aptameric 2′-fluoro RNA oligomer (Scr) of the same length as 2′-fluoro RNA aptamers 21-2 and 3-4. We also employed a therapeutic monoclonal antibody, secukinumab (Cosentyx, Novartis), as a control reference inhibitor of the IL-17A.

**Figure 1:**
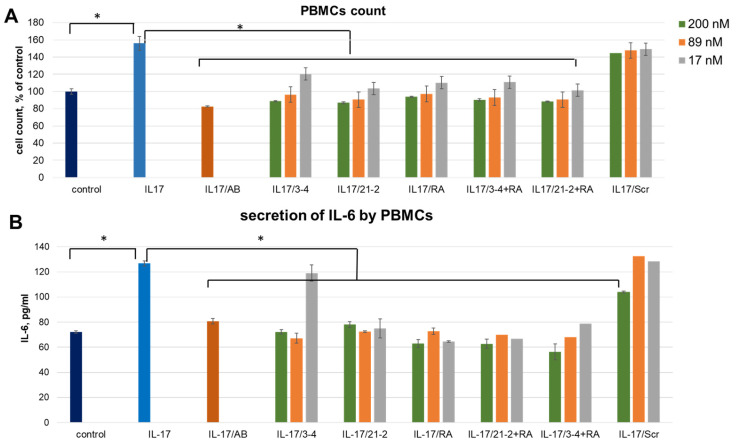
An impact of IL-17A and IL-17RA specific aptamers on IL-17A-stimulated cell proliferation (**A**) and IL-6 secretion (**B**) in PBMC. The aptamers and their combinations are designated as follows: IL-17/AB—IL-17A + monoclonal antibody (secukinumab, 10 µM); IL-17/3-4—IL-17A + aptamer 3-4; IL-17/21-2—IL-17A + aptamer 21-2; IL-17/RA—IL-17A + aptamer RA10-6; IL-17/21-2 + RA—IL-17A + aptamers 21-2 and RA10-6; IL-17/3-4 + RA—IL-17 + aptamers 3-4 and RA10-6; IL-17/Scr—IL-17A + non-aptamer control Scr. The concentrations of aptamers and control oligonucleotide are given in the legend. *n* = 3, *—*p* < 0.05.

A combined treatment of PBMC by the reference anti-IL-17A monoclonal antibody (secukinumab) resulted in the same level of proliferation as for control cells, thus showing a full blockage of the IL-17A stimulation (Figure 1A). The same inhibiting effect was observed after adding IL-17A pre-incubated with aptamers. We registered an obvious concentration dependence of the inhibition level for all three aptamers. In contrast, a non-aptamer control oligonucleotide, Scr, did not influence proliferation, thus proving the specific action of the aptamers. IL-6 secretion by IL-17A-stimulated PBMC showed the same dependencies as proliferative activity (Figure 1B). In this case, non-aptamer control Scr at the maximal concentration (200 nM) showed a slight inhibitory effect, much weaker than that for specific aptamers. Interestingly, in both experiments, simultaneous addition of anti-ILA and anti-IL17A aptamers did not provide any synergistic effect.

### 2.2. Characterization of FLS from axSpA and RA Patients

**Figure 2:**
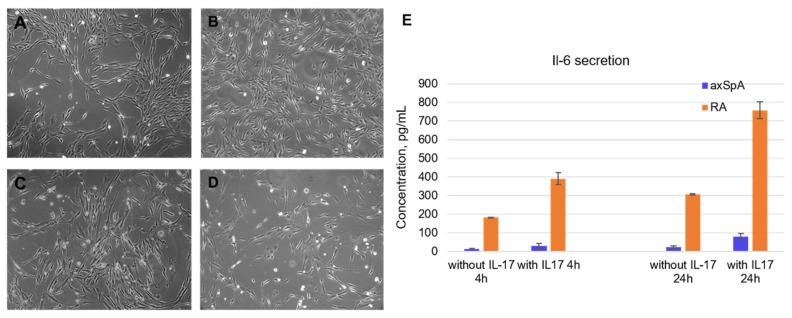
Characterization of fibroblast-like synoviocytes isolated from synovial fluid of axSpA and RA patients. (**A**). Morphology of axSpA FLS at the 1st passage. (**B**). Morphology of RA FLS at the 1st passage. (**C**). Morphology of axSpA FLS at the 4th passage. (**D**). Morphology of RA FLS at the 4th passage. (**E**). Basal and IL-17A-stimulated IL-6 secretion by FLS from RA and axSpA patients (3rd passage). All light microscopy images are obtained at ×20 magnification.

Fibroblast-like synoviocytes were isolated from synovial fluid samples taken from axApA and RA patients. The experiment started with the choice of cell lines for testing the aptamers. It was found that the level of IL-6 secretion significantly decreases with the passage number, becoming completely depleted by passages 5–6. Moreover, FLSs from RA patients demonstrated higher levels of IL-6 secretion than axSpA-specific FLS. The axSpA cell lines of the 3rd–4th passage were characterized by a basal IL-6 secretion of 14.4 ± 1.8 pg/mL after 4 h of cultivation and 24.8 ± 6.7 pg/mL after 24 h. After IL-17A stimulation (50 ng/mL), the IL-6 levels elevated up to 29.4 ± 13.6 pg/mL and 78.6 ± 18.9 pg/mL after 4 and 24 h, respectively. Of note, FLS from RA patients showed an order of magnitude higher IL-6 secretion: Basal levels were 181.5 ± 2.2 pg/mL after 4 h and 306.3 ± 2.3 pg/mL after 24 h. IL-17A stimulation elevated these values to 390.7 ± 32.5 pg/mL and 756.5 ± 44.7 pg/mL after 4 h and 24 h, respectively (Figure 2E).

To characterize IL-17A suppression by the aptamers, we studied their effects on FLS cells related to both pathologies.

Morphologically, the isolated FLS possessed a fibroblast-like spindle form; the mean double time of the population was 24 h (Figure 2A–D). All cell lines were characterized on their cell cycle, basal IL-6, and MMP-13 secretion depending on the passage number and donor. For subsequent experiments, we have chosen cell lines meeting the criteria for assessment of anticytokine therapeutics, namely those with moderate IL-6 basal secretion and a prominent response to IL-17A stimulation.

### 2.3. Evaluation of Inhibiting Effects of Anti-IL17A and Anti-IL-17RA Aptamers of IL-17A Stimulation of FLS from axSpA and RA Patients

As was reported in [36], in rheumatic inflammatory joint diseases, the density of IL-17 receptors on synoviosytes significantly increases. This, in turn, changes the character of the response to IL-17 and affects the efficacy of therapeutic inhibition of the cytokine. Taking into account an elevated receptor density and a greater level of response that could be more resistant to target inhibitors, we used only a high concentration of the aptamers (200 nM).

FLS cell lines possessed high proliferative potential (44.7 ± 1.65% of the cells in S and G2/M phases of the cell cycle), and IL-17A did not significantly influence proliferation. Therefore, we did not use this parameter for the evaluation of the inhibiting activity.

**Figure 3:**
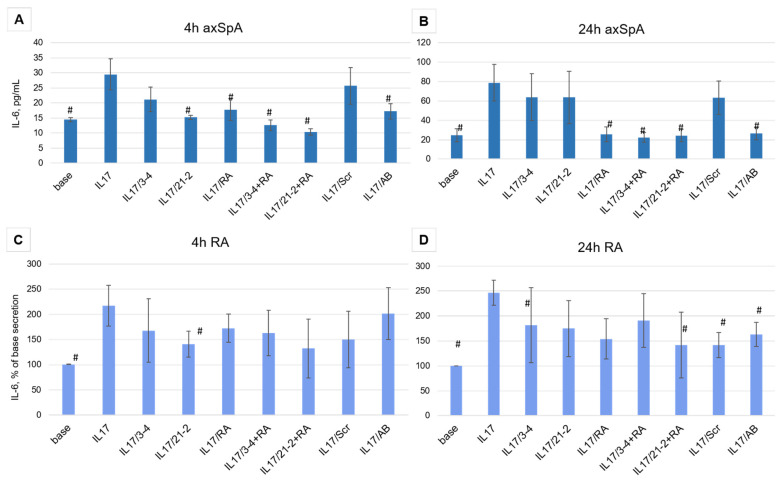
Inhibition of IL-17A stimulated secretion of IL-6 by the aptamers in FLS cell cultures from axSpA (panels (**A**,**B**)) and RA (panels (**C**,**D**)) patients. IL-6 levels were measured after 4 and 24 h of incubation. The aptamers and their combinations are designated as follows: IL-17/AB—IL-17A + monoclonal antibody (secukinumab, 10 µM); IL-17/3-4—IL-17A + aptamer 3-4; IL-17/21-2—IL-17A + aptamer 21-2; IL-17/RA—IL-17A + aptamer RA10-6; IL-17/21-2 + RA—IL-17A + aptamers 21-2 and RA10-6; IL-17/3-4 + RA—IL-17 + aptamers 3-4 and RA10-6; IL-17/Scr—IL-17A + non-aptamer control Scr. *n* = 6. #—*p* < 0,05 as related to level the IL-17 induced IL-6 secretion.

Aptamer treatment of FLS cells from axSpA patients showed different efficacy depending on the time of incubation. After 4 h of the IL-17A stimulation, both anti-IL-17A 2′-F-RNA aptamers 3-4 and 21-2 and the anti-IL-17RA DNA aptamer RA10-6 showed inhibition of IL-17A activity nearly to the same extent as the control antibody (Figure 3A). A combination of anti-cytokine and anti-receptor aptamers demonstrated a synergistic effect, providing stronger suppression of IL-17A signaling than both aptamers alone. However, after 24 h, the inhibiting activities of 3-4 and 21-2 alone were much lower than that of secukinumab. At the same time, RA10-6 and its combinations with anti-IL-17A aptamers showed nearly the same high efficacy, equal to that of secukinumab (Figure 3B). Meanwhile, in FLS from RA patients, all these effects were much weaker, and even the reference monoclonal anti-IL-17A antibody secukinumab did not lower IL-17-stimulated secretion of IL-6 to the basal level (Figure 3C,D).

**Figure 4:**
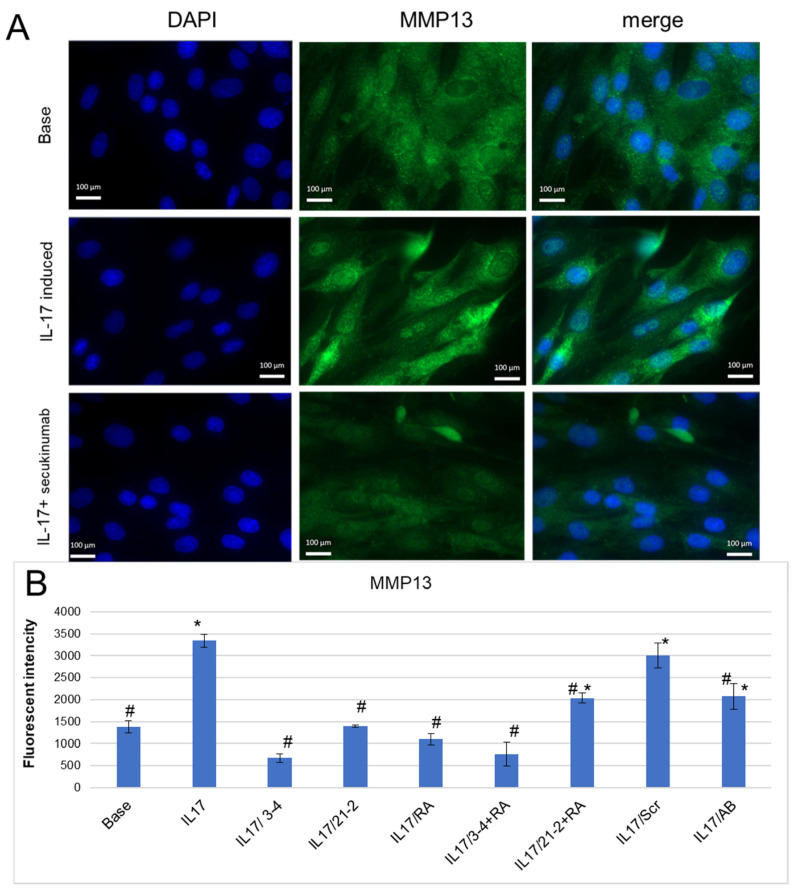
Inhibition of IL-17A-stimulated secretion of MMP-13 by the aptamers in FLS cell cultures from axSpA. (**A**). A representative fluorescent microphotograph of FLS after IL-17A stimulation. Magnification ×40. DAPI-stained nuclei, blue signal. Fluorescein-labeled antibody to MMP13, green signal. (**B**). MMP-13 secretion levels of FLS evaluated by flow cytometry. The aptamers and their combinations are designated as follows: IL-17/AB—IL-17A + monoclonal antibody (secukinumab, 10 µM); IL-17/3-4—IL-17A + aptamer 3-4; IL-17/21-2—IL-17A + aptamer 21-2; IL-17/RA—IL-17A + aptamer RA10-6; IL-17/21-2 + RA—IL-17A + aptamers 21-2 and RA10-6; IL-17/3-4 + RA—IL-17 + aptamers 3-4 and RA10-6; IL-17/Scr—IL-17A + non-aptamer control Scr. *n* = 3. *—*p* < 0.05 as related to basal secretion of MMP-13; #—*p* < 0.05 as related to IL-17A stimulated secretion of MMP-13.

A mechanism of tissue degradation in inflammatory arthropathies includes the proteolysis of structural molecules in the extracellular matrix (ECM) associated with elevated levels of remodeling enzymes, particularly matrix metalloproteinases (MMPs) [37,38,39]. In our experiments, IL-17 reliably enhanced MMP-13 secretion by FLS (Figure 4B), as it was shown by fluorescent microscopy (Figure 4A) and flow cytometry (Figure 4B) with staining by anti-MMP-13 antibodies. The use of secukinumab for IL-17A inhibition lowered MMP-13 to the basal level (Figure 4A,B). Anti-IL-17A and anti-IL-17RA aptamers also decreased MMP-13 secretion and demonstrated an even greater extent of suppression of IL-17A signaling than secukinumab (Figure 4B). Among them, anti-IL-17A 2′-F-RNA aptamer 3-4 was the most effective, both alone and in combination with RA10-6.

To assess the specificity of the IL-17 aptamers, we investigated their potential off-target effect on the inflammatory response initiated by another key pro-inflammatory cytokine, tumor necrosis factor-α (TNF-α). Stimulation of FLS cells from axSpA patients with TNF-α, as in the case of IL-17-induced stimulation, produced an increase in MMP13 levels. However, all tested aptamers (3-4, 21-2, or their combination with the receptor-targeting aptamer RA10-6) did not show any statistically significant inhibitory effect on TNF-α-induced MMP13 secretion (*p* > 0.05) (Supplementary Materials, Figure S2).

## 3. Discussion

In the present study, we employed two different in vitro models with effector cells for IL-17A: peripheral blood mononuclear cells (PBMCs) and type B fibroblast-like synoviocytes from axSpA and RA patients. The PBMC fraction includes lymphocytes, monocytes, natural killers (NK cells), dendritic cells, and progenitor cells. All of them play an active role in inflammation, particularly through IL-17 activation, which is manifested in their recruitment, stimulation of proliferation, and secretion of IL-6, IL-8, and GM-CSF.

Besides immune cells, the receptor of IL-17 is widely expressed and mediates its effects through a number of cells not related to the immune system. Endothelial and epithelial cells are especially sensitive to IL-17 [40]. In spondylitis and rheumatoid arthritis, the main symptomatic manifestation is joint damage through an inflammatory process that involves resident cells, fibroblast-like synoviocytes [41]. They actively maintain inflammation and destruction of bone and cartilage tissues through the secretion of pro-inflammatory cytokines, namely IL-6, and metalloproteinases [42]. Moreover, FLSs are very sensitive to IL-17, which further activates their secretory activity. These features made them a relevant cell model for preclinical evaluation of targeted therapeutics in rheumatology [43]. Therefore, we also employed FLS as a pathogenetic in vitro model for testing anti-cytokine aptamers.

A strategy of combined action on both IL-17 and its receptor for better therapeutic response appears to be rather promising, since the receptor was shown to possess an autonomic activity [44], but such schemes have not yet been applied in clinics. In our study, we examined the effects of anti-IL17A and anti-IL17RA aptamers used either separately or in combinations.

We started from the evaluation of the inhibitory activity of the aptamers on PBMC from a healthy donor. In this cell model, all aptamers in the concentration range of 17–200 nM suppressed IL-17A-stimulated cell proliferation and IL-6 secretion, showing the efficacy comparable to that of secukinumab, a reference anti-IL-17A therapeutic antibody. Notably, a non-aptamer scrambled oligonucleotide Scr did not demonstrate any significant effect, thus proving the specific action of the aptamers. The combined action of cytokine- and receptor-targeted aptamers did not result in a prominent synergetic effect, allowing us to presume that the blockage of any of these players is sufficient to suppress signaling in healthy PBMCs.

The most interesting and clinically relevant observations were made on FLS cell lines from axSpA and RA patients. We revealed that the response to the IL-17 inhibition depends on the time of incubation with IL-17A (with or without aptamers) and on the particular nosology. In axSpA FLS, the receptor-targeted aptamer RA10-6, either alone or in combination with an anti-cytokine aptamer, efficiently suppressed IL-6 secretion after 4h, showing nearly the same effect as secukinumab. However, the effect of anti-IL17A aptamers almost disappeared after 24 h, while the receptor-targeting aptamer continued to prevent the pro-inflammatory action of the cytokine.

In contrast, for FLS cells from RA patients, we observed a much weaker effect of all inhibitors, including the monoclonal antibody. This finding is consistent with the literature data on the heterogeneity of IL-17 receptors and particularities of intracellular signaling in different immunoinflammatory rheumatic diseases [36,45,46]. Moreover, in clinical trials, anti-IL-17A biologics showed only modest efficacy in RA (see, e.g., review [15]. It is likely that in rheumatoid arthritis, the impact of other pro-inflammatory pathways becomes more important, making the blockage of only IL-17A less effective. Otherwise, RA-derived FLS may expose a higher density of IL-17 receptors, thus demanding higher concentrations of targeted blockers. These suggestions would be examined in our further studies.

We also consider important the finding that aptamers can inhibit IL-17A-stimulated secretion of the matrix metalloproteinase MMP-13 that plays a key role in type II collagen degradation within joint cartilage. MMP-13 inhibition is an important therapeutic mechanism for preventing structural damage. According to our results, anti-IL-17A and anti-IL-17RA aptamers return the MMP-13 level to a basal value, which highlights their potential not only for inflammation control but also for the protection of joint tissues from destruction. It is also worth noting that we observed the inhibition of cytokine-induced MMP-13 production only for IL-17-stimulated cells, while TNF-α-induced MMP-13 production was not influenced by anti-IL17 aptamers. These results indicate the absence of non-specific immunomodulatory action of the aptamers and allow us to attribute their anti-inflammatory activity to the specific inhibition of the IL-17 axis.

## 4. Materials and Methods

### 4.1. Chemicals and Reagents

A controlled pore glass support (CPG) derivatized with the first nucleoside, 5′,N-protected 2′-O-TBDMS-ribophoshporamidites (A and G), and 5′,N-protected 2′-deoxyribophoshporamidites were purchased from Glen Research Inc. (Sterling, VA, USA). The 5′,N-protected 2′-deoxy-2′-fluoro pyrimidine phosphoramidites were purchased from ChemGene Corp. (Wilmington, MA, USA). Active IL-17 and active TNFα recombinant proteins were purchased from Cloud-Clone Corp. (Wuhan, China).

### 4.2. Synthesis of Oligonucleotides

The 2′-F-RNA aptamers, DNA aptamer, and control scrambled 2′-F-RNA oligonucleotide (Table 1) were synthesized by the solid-phase phosphoramidite method at the 0.4 µmol scale on an automated DNA/RNA synthesizer ASM-800 (Biosset, Novosibirsk, Russia) by the protocols optimized for the instrument. Synthesis, deprotection, and isolation procedures were performed as described in [47].

### 4.3. Cell Lines

The studies involving biological materials from healthy donors and patients were conducted in accordance with the Declaration of Helsinki and were approved by the RICEL Local Ethical Committee (protocol No. 183, 11 August 2023).

Cytotoxicity and activity of anti-IL-17 aptamers were studied using PBMCs (peripheral blood mononuclear cells) and fibroblast-like synoviocytes (FLSs). PBMCs were isolated from the peripheral blood of a healthy donor by Ficoll density gradient centrifugation according to the protocol in [41] with minor changes. Briefly, 4 mL of human venous blood samples was collected in heparinized vials (Vacuette, 6 mL, Greiner Bio-One, Kremsmünster, Austria). Each sample was supplied with 4 mL PBS. Then, 3 mL of the Ficoll solution (Paneco, Moscow, Russia) was placed in a 15 mL centrifuge tube, and the blood sample was gently layered on top of the Ficoll solution and centrifuged for 15 min at 400× *g* at room temperature. PBMCs were collected from the whitish meniscus between the plasma and medium and washed twice with RPMI. Then, the cells were cultured in an RPMI nutrient medium supplied with 10% calf serum (Gibco, ThermoFisher, Waltham, MA, USA) and 1% penicillin streptomycin (Gibco, ThermoFisher, USA). Cells were seeded in 96-well culture plates at a density of 5 × 10^3^ cells/100 µL per well.

After 24 h, the cells were treated with a solution containing IL-17 (50 ng/mL) and aptamers (17 nM, 89 nM, and 200 nM). IL-17 was preincubated with aptamers and with secukinumab (50 ng/mL) as a reference anti-interleukin-17A monoclonal antibody for 60 min before adding to cells. Fresh culture medium was added to the control cells. Then, cells were cultivated at 37 °C under a 5% CO_2_ humidified atmosphere for 24 h.

FLSs were isolated from the synovial fluid samples obtained during the therapeutic joint biopsy of axSpA (*n* = 2) and RA patients (*n* = 2). Each patient signed a corresponding informed consent form. The synovial fluid was centrifuged at 300× *g*, and the cell pellet was cultured in Dulbecco’s modified Eagle’s medium (Gibco; Thermo Fisher Scientific) containing 10% fetal bovine serum (Gibco; Thermo Fisher Scientific) and 1% penicillin streptomycin (Gibco; Thermo Fisher Scientific) at 37 °C in the presence of 5% CO_2_ for 24 h; then, non-adherent cells were removed. After obtaining a monolayer with 70–80% confluence, cells were reseeded with the use of Trypsin-EDTA (0.25%) (Gibco, Thermo Fisher Scientific). The resulting lines were tested for mycoplasma contamination; then, their immunophenotype (CD 45^−^, 44^+^, 73^+^, 90^+/−^) and the basal and IL-17A-induced secretion of IL-6 and MMP-13 were examined. To ensure robustness and reproducibility, all experiments were performed in triplicate on three independent days using cells from these subsequent passages (P3–P4). As a control cell line, we employed dermal fibroblasts from donors with no rheumatic diseases.

When cell confluency reached ~80%, cells were seeded in 96-well culture plates at a density of 5 × 10^3^ cells/100 µL/well. For fluorescent microscopy, we used a 96-well µ-Plate (iBiDi). After 24 h, the cells were treated with a solution containing either IL-17A or TNF-ɑ alone (concentration of 50 ng/mL) or the same concentration of IL-17A or TNF-α combined with aptamers (in the maximal concentration (200 nM) or secukinumab (50 ng/mL)) as a reference anti-IL-17A monoclonal antibody. Prior to adding to cells, IL-17A was preincubated with aptamers or secukinumab for 60 min. Fresh culture medium was added to control cells. After treatment, cells were cultivated at 37 °C under a 5% CO_2_ humidified atmosphere for 4 h, 24 h, and 72 h depending on the further goal. IL-6 secretion was determined after 4 h and 24 h and MMP13 secretion after 24 h, and the effect on cell viability was evaluated after 72 h.

### 4.4. Cell Viability and Proliferation

For adherent FLS cultures, cell viability and proliferative activity were determined by cell counting using the DNA-intercalating dye Hoechst 33442. For PBMC suspension cultures, which contain a fraction of adherent cells (e.g., monocytes/macrophages), the entire cell pool was collected for analysis: Non-adherent cells were combined with the adherent fraction after its detachment by trypsinization. Total cell counts were then determined with a C100 automated cell counter (RWD Life Science, Shenzhen, China). Each parameter was assessed in three independent biological replicates, each with technical triplicates.

### 4.5. Measurement of the IL-6 Secretion Levels of FLS

The level of IL-6 secreted by cells in the culture medium in response to IL-17A stimulation after 4 h and 24 h was measured using the ELISA kit (A-8768, Vector Best, Novosibirsk, Russia) according to the manufacturer’s recommendations.

### 4.6. Evaluation of the MMP-13 Secretion Levels of FLS

The levels of MMP-13 secretion of FLS were examined by fluorescent microscopy (Axio Imager A2, Zeiss, Oberkochen, Germany) and flow cytometry (FACSCanto, BD, San Jose, CA, USA) after 72 h of incubation. The cells were incubated with IL-17A/aptamers/secukinumab for 24 h according to the protocol described in Section 4.2. The analysis was performed with the use of anti-MMP13 antibodies (PAA099Hu01, CloudClone, Wuhan, China, https://www.cloud-clone.com/products/PAA099Hu01.html, accessed on 23 January 2026), secondary FITC-labeled antibodies (ab150077, goat anti-mouse, Abcam, Cambridge, UK) for fluorescent microscopy, and PE-labeled anti-MMP13 (LAA099Hu44, CloudClone, China) for flow cytometry. Before staining, the cells were fixed with 4% paraformaldehyde and permeabilized with a 0.5% Triton X-100 solution in PBS. Staining was performed according to the manufacturer’s protocol.

All measurements were carried out in at least three replications.

### 4.7. Statistical Analysis

For all results, the mean values and associated standard deviations were calculated. For statistical analysis, we used Statistica 8 software. *p*-value ≤ 0.05 was considered statistically significant. The Shapiro–Wilk test was used to control the normal distribution of all variables. Since the obtained data did not show a normal distribution, a nonparametric test was appropriate; namely, the nonparametric Mann–Whitney U test was used for comparing two groups, and the Kruskal–Wallis test was used for comparing more than two groups.

## 5. Conclusions

To summarize, our study demonstrates that 2′-F-RNA and DNA aptamers targeting either IL-17A or its receptor can effectively suppress key pro-inflammatory and tissue-destructive responses in vitro. Using patient-derived fibroblast-like synoviocytes as a pathologically relevant model, we show that the aptamers inhibit the IL-17A-induced secretion of IL-6 and MMP-13, and their efficacy is time-dependent and differs between axSpA and RA. Interestingly, the receptor-targeting aptamer RA10-6 exhibited a more sustained inhibitory effect. Our study provides the first example of an in vitro functional assay in human cell models of rheumatic disease that establishes a specific efficacy of the aptamers and shows their potential to become an alternative to therapeutic antibodies. In particular, simultaneous use of aptamers targeting both IL-17A and IL-17RA resulted in a synergistic effect that made this combination even better than the reference antibody, secukinumab.

The subsequent studies will focus on the optimization of aptamer structure to further improve their affinity and in vivo half-life and on preclinical investigations of their efficacy and safety on relevant animal models.

## Figures and Tables

**Table 1:** Nucleotide sequences of aptamers targeting IL-17A and IL-17RA and a control scrambled oligonucleotide.

Aptamer	Nucleotide Sequence, 5′->3′
Apt21-2	GGU^F^CU^F^AGC^F^C^F^GGAGGAGU^F^C^F^AGU^F^AAU^F^C^F^GGU^F^AGAC^F^C^F^T_inv_
Apt3-4	GGAU^F^AGC^F^GAAGU^F^CAU^F^U^F^GAGC^F^GC^F^C^F^T_inv_
RA10-6	d(GCGGAATTCTAATACGACTCACTATAGGGAACAGTCCGAGCCCTTGGA TCACCATAGTCGCTAGTCGAGGCTGGGTCAATGCGTCATA)
Scr	AC^F^U^F^GGU^F^AU^F^GU^F^C^F^GAGC^F^C^F^AAC^F^AAU^F^C^F^GAU^F^AC^F^C^F^AAGAC^F^U^F^AAGA [47]

C^F^—2′-fluorocytidine; U^F^—2′-fluorouridine; T_inv_—3′-terminal thymidine residue attached by the 3′-3′-phosphodiester linkage.

## Data Availability

The original contributions presented in this study are included in the article and Supplementary Materials. Further inquiries can be directed to the corresponding authors.

## Supplementary Materials

The following supporting information can be downloaded at https://www.mdpi.com/article/10.3390/ph19020238/s1. Figure S1. The effect of aptamers on the proliferation and viability of peripheral blood mononuclear cells (PBMCs) and fibroblast-like synoviocytes (FLS). The aptamers themselves do not exhibit inhibitory effects on the viability of the cells (PBMCs and FLS) used for testing their ability to suppress IL-17-induced secretion of IL-6 and MMP-13. Figure S2. MMP-13 secretion levels of FLS evaluated by flow cytometry in FLS from axSpA patients. Cells were stimulated either by TNFα or by IL-17A. The aptamers and their combinations are designated as follows: TNF–TNF-α; TNF/3-4–TNF-ɑ + aptamer 3-4; TNF/21-2-TNF-ɑ + aptamer 21-2; TNF/21-2+RA–TNF-ɑ + aptamers 21-2 and RA10-6; TNF/3-4+RA–TNF-ɑ + aptamers 3-4 and RA10-6; TNF/Scr—TNF-ɑ + non-aptamer control Scr; IL-17/AB—IL-17A + anti-IL17A monoclonal antibody (secukinumab, 10 µM); IL-17/3-4—IL-17A + aptamer 3-4; IL-17/21-2—IL-17A + aptamer 21-2; IL-17/21-2+RA—IL-17A + aptamers 21-2 and RA10-6; IL-17/3-4+RA—IL-17 + aptamers 3-4 and RA10-6; IL-17/AB—IL-17A + monoclonal antibody (secukinumab, 10 µM); IL-17/Scr—IL-17A + non-aptamer control Scr. *n* = 3. TNF-α concentration was 50 ng/mL, IL-17A concentration was 50 ng/mL. *—*p* < 0.05 as related to basal secretion of MMP-13, #—*p* < 0.05 as related to TNF-a or IL-17A stimulated secretion of MMP-13

## Abbreviations

axSpA	Axial spondyloarthritis
COPD	Chronic obstructive pulmonary disease
DAPI	4′,6-diamidino-2-phenylindole DNA stain
EDTA	Ethylenediaminetetraacetic acid
FITC	Fluorescein isothiocyanate
FLS	Fibroblast-like synoviocyte
GM-CSF	Granulocyte-macrophage colony-stimulating factor
IL-6	Interleukin-6
IL-17	Interleukin-17
IL-17R	IL-17 receptor
MMP	Matrix metalloproteinase
NK	Natural killer cells
PBMC	Peripheral blood mononuclear cell
PBS	Phosphate saline buffer
RA	Rheumatoid arthritis
RORγt	RAR-related orphan receptor-γ
