# Supplementary material for: Aptamers Targeting IL17A and Its Receptor Suppress IL17 Signaling in Different Cell Types
Source: Pharmaceuticals (Basel). 2026 Jan 29;19(2):238. doi: 10.3390/ph19020238 (PMC12943235; doi:10.3390/ph19020238)
Supplement: Supplementary file 1 [file pharmaceuticals-19-00238-s001.zip › pharmaceuticals-4012748-supplementary.pdf]

The effect of oligonucleotide aptamers on the viability of PBMC and FLS from patients

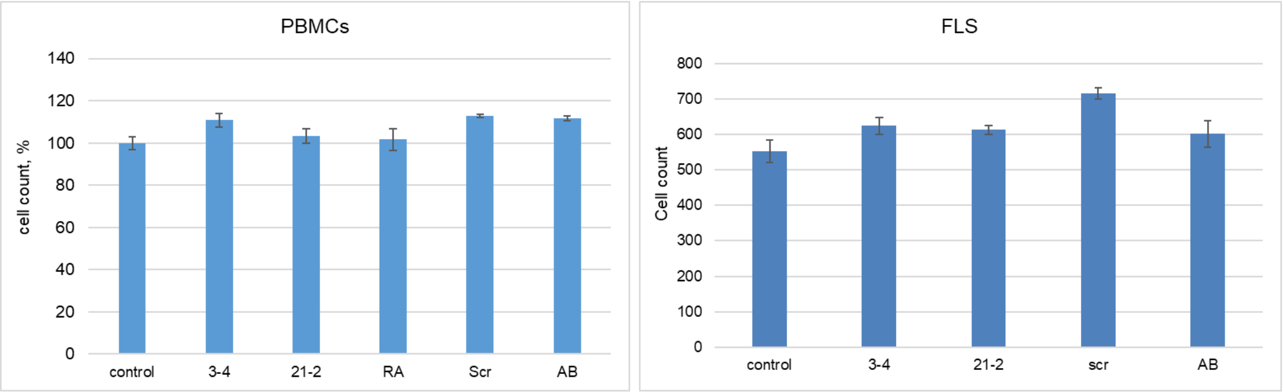

Figure S1. The effect of aptamers on the proliferation and viability of peripheral blood mononuclear cells (PBMCs) and fibroblast-like synoviocytes (FLS).The aptamers themselves do not exhibit inhibitory effects on the viability of the cells (PBMCs and FLS) used for testing their ability to suppress IL-17-induced secretion of IL-6 and MMP-13.

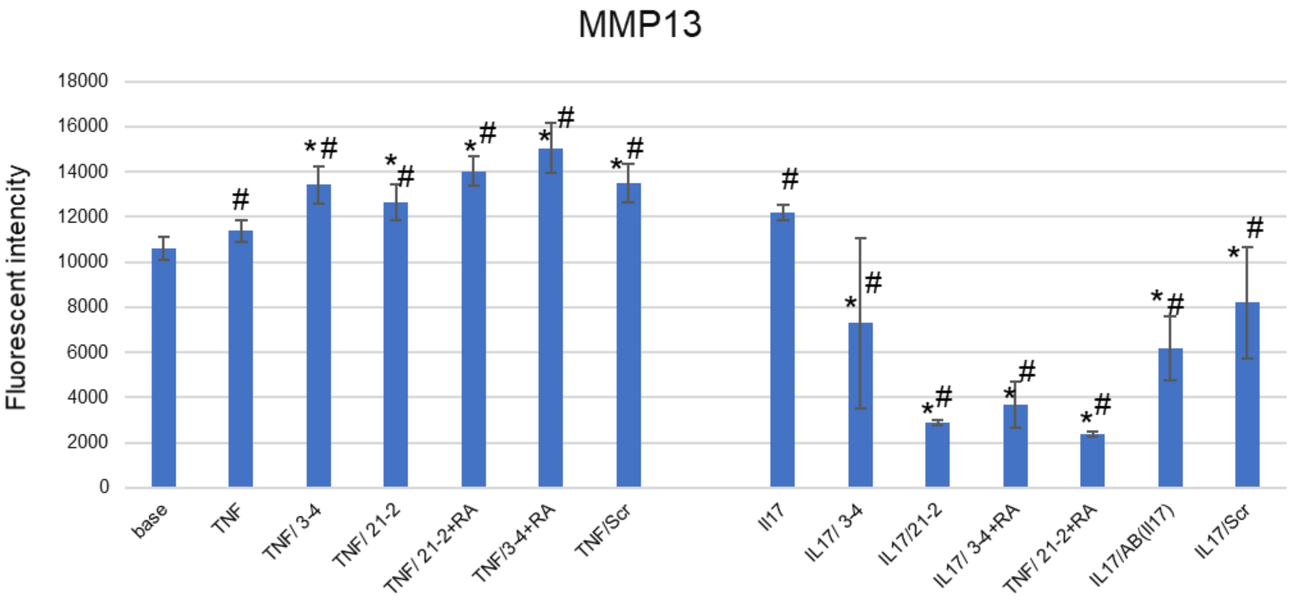

Figure S2. MMP-13 secretion levels of FLS evaluated by flow cytometry in FLS from axSpA patients. Cells were stimulated either by TNFα or by IL-17A. The aptamers and their combinations are designated as follows: TNF – TNF-α; TNF/3-4 – TNF-α + aptamer 3-4; TNF/21-2 - TNF-α + aptamer 21-2; TNF/21-2+RA – TNF-α + aptamers 21-2 and RA10-6; TNF/3-4+RA – TNF-α + aptamers 3-4 and RA10-6; TNF/Scr — TNF-α + non-aptamer control Scr; IL-17/AB — IL-17A + anti-IL17A monoclonal antibody (secukinumab, 10 µM); IL-17/3-4 — IL-17A + aptamer 3-4; IL-17/21-2 — IL-17A + aptamer 21-2; IL-17/21-2+RA — IL-17A + aptamers 21-2 and RA10-6; IL-17/3-4+RA — IL-17 + aptamers 3-4 and RA10-6; IL-17/AB — IL-17A + monoclonal antibody (secukinumab, 10 µM); IL-17/Scr — IL-17A + non-aptamer control Scr. n=3. TNF-α concentration was 50 ng/mL, IL-17A concentration was 50 ng/mL. * - $p<0,05$ as related to basal secretion of MMP-13, # - $p<0,05$ as related to TNF-a or IL-17A stimulated secretion of MMP-13.
